# Salt Stress Induces Paramylon Accumulation and Fine-Tuning of the Macro-Organization of Thylakoid Membranes in *Euglena gracilis* Cells

**DOI:** 10.3389/fpls.2021.725699

**Published:** 2021-11-16

**Authors:** Sai Divya Kanna, Ildikó Domonkos, Tímea Ottília Kóbori, Ágnes Dergez, Kinga Böde, Sarolta Nagyapáti, Ottó Zsiros, Renáta Ünnep, Gergely Nagy, Gyözö Garab, László Szilák, Katalin Solymosi, László Kovács, Bettina Ughy

**Affiliations:** ^1^Institute of Plant Biology, Biological Research Centre, Eötvös Loránd Research Network, Szeged, Hungary; ^2^Doctoral School of Biology, University of Szeged, Szeged, Hungary; ^3^Division for Biotechnology, Bay Zoltán Nonprofit Ltd. for Applied Research, Szeged, Hungary; ^4^Institute for Solid State Physics and Optics, Wigner Research Centre for Physics, Eötvös Loránd Research Network, Budapest, Hungary; ^5^European Spallation Source ESS ERIC, Lund, Sweden; ^6^Laboratory for Neutron Scattering and Imaging, Paul Scherrer Institute, Villigen PSI, Villigen, Switzerland; ^7^Neutron Scattering Division, Oak Ridge National Laboratory, Oak Ridge, TN, United States; ^8^Faculty of Science, University of Ostrava, Ostrava, Czechia; ^9^Szilak Laboratory Ltd., Szeged, Hungary; ^10^Department of Plant Anatomy, ELTE Eötvös Loránd University, Budapest, Hungary

**Keywords:** salt stress, *Euglena gracilis*, paramylon, microalgae, photosynthesis

## Abstract

The effects of salt stress condition on the growth, morphology, photosynthetic performance, and paramylon content were examined in the mixotrophic, unicellular, flagellate *Euglena gracilis*. We found that salt stress negatively influenced cell growth, accompanied by a decrease in chlorophyll (Chl) content. Circular dichroism (CD) spectroscopy revealed the changes in the macro-organization of pigment-protein complexes due to salt treatment, while the small-angle neutron scattering (SANS) investigations suggested a reduction in the thylakoid stacking, an effect confirmed by the transmission electron microscopy (TEM). At the same time, the analysis of the thylakoid membrane complexes using native-polyacrylamide gel electrophoresis (PAGE) revealed no significant change in the composition of supercomplexes of the photosynthetic apparatus. Salt stress did not substantially affect the photosynthetic activity, as reflected by the fact that Chl fluorescence yield, electron transport rate (ETR), and energy transfer between the photosystems did not change considerably in the salt-grown cells. We have observed notable increases in the carotenoid-to-Chl ratio and the accumulation of paramylon in the salt-treated cells. We propose that the accumulation of storage polysaccharides and changes in the pigment composition and thylakoid membrane organization help the adaptation of *E. gracilis* cells to salt stress and contribute to the maintenance of cellular processes under stress conditions.

## Introduction

The growth, productivity, and survival of photosynthetic organisms are significantly affected by abiotic stresses. Ever-changing environmental conditions can affect cell morphology, division, membrane composition, and cellular metabolism. Salinity is one of the major abiotic stresses of photosynthetic organisms. Aquatic organisms especially can be exposed to a wide range of salinity in nature, affecting their growth and even their survival (Velasco et al., 2019). Microalgae are capable of responding to the changing environments with physiological and developmental changes and adjusting their metabolic pathways and the organization of their thylakoid membranes. Salt stress can disturb cellular homeostasis leading to molecular damage, growth arrest, and even cell death. In most cases, salt stress induces ionic and osmotic imbalance followed by oxidative damage caused by reactive oxygen species (ROS), which can lead to the loss of photosynthetic efficiency. The salt treatment of *Chlamydomonas reinhardtii* (*C*. *reinhardtii*) cells has been shown to lower the growth efficiency of the culture, and induced motility loss, palmelloids formation, and reduced cell size (Neelam and Subramanyam, 2013). Salt stress in *Dunaliella salina* induced continuous alteration of cell size (Fu et al., 2014). In higher plants, the activity of both photosystems was impaired under salt stress leading to a reduced electron transport rate (ETR) (Tiwari et al., 1998; Parida et al., 2003). Increased sodium ion concentration inhibited the electron transport activity on both the donor and acceptor sides of the photosystem II (PSII) (Verma and Mohanty, 2000), whereas the cyclic electron flow around the photosystem I (PSI) was augmented under salt stress in cyanobacteria (Gilmour et al., 1985; Joset et al., 1996). Salt treatment can affect the protein composition of the photosynthetic apparatus in higher plants (Kuwabara and Murata, 1982; Murata et al., 1992), cyanobacteria (Sudhir et al., 2005), and microalgae (Subramanyam et al., 2010; Neelam and Subramanyam, 2013). When the plants are exposed to salt stress, protective mechanisms are activated to prevent or alleviate the damage and re-establish the homeostasis of the cells, which thus restores other cellular processes and growth (Zhu, 2001). One of the adaptation responses is the accumulation of compatible solutes. The compatible solutes protect the cells from harsh conditions by osmoregulation, scavenging ROS, and maintaining membrane integrity and enzyme activity (Yancey, 1994). Increased glycerol accumulation has been recorded upon elevated salt concentration in *C. reinhardtii* (León and Galván, 1994), *Dunaliella parva* (Ben-Amotz and Avron, 1973), and *Chlorella autotrophica* (Ahmad and Hellebust, 1984). Exploring the adaptation of biotechnologically important microalgal strains to higher salt concentrations is highly important, opening new vistas for using salty or brackish waters.

*Euglena gracilis* is a mixotrophic microalgal species of unicellular freshwater flagellate protists, having high biotechnological potential (Krajčovič et al., 2015). *E. gracillis* is a perfect source of numerous valuable compounds (O'neill et al., 2015), such as paramylon (Barsanti et al., 2001; Sun et al., 2018), wax esters (Inui et al., 1982; Zimorski et al., 2017), polyunsaturated fatty acids, α-tocopherol (vitamin E), biotin (vitamin B_7_), and proteins (Gissibl et al., 2019). *E. gracilis* can be grown autotrophically, mixotrophically, or heterotrophically (Wang et al., 2018). Their plastids are acquired by secondary endosymbiosis (Lefort-Tran et al., 1980). A significant difference between the thylakoid membrane of microalgae and higher plants is that the microalgae generally possess more loosely stacked membrane systems. The chloroplast ultrastructure of *E. gracilis* was shown to be similar to that of dinoflagellates (Gibbs, 1960). Importantly, both photosystems of *E. gracilis* seem to use a common antenna system comprising both light harvesting complex-I (LHCI) and light harvesting complex-II (LHCII), which can participate in spill-over of the excitation energy from PSII to PSI contrary to that of other microalgae and higher plants (Doege et al., 2000). Furthermore, the pigment composition of *E. gracilis* is different from that of plants and green algae. *E. gracilis* contains β-carotene and xanthophylls, such as neoxanthin (Nx) and diadinoxanthin (Ddx), but pigments of the classical violaxanthin cycle are absent (Doege et al., 2000). The presence of Ddx in *Euglena* was proposed to stabilize the LHCs, similar to lutein in the higher plants (Brandt and Wilhelm, 1990). *E. gracilis* can tolerate variations of different environmental factors, such as pH, temperature, oxygen concentration, and salinity (Richter et al., 2003). The cells are able to regulate their position in the water column by photo- and gravitaxis. Salinity can influence these movements in a concentration-dependent way (Richter et al., 2003). High-salt stress can negatively affect the photosynthetic performance of *E. gracilils* cells and, under osmotic stress, trehalose accumulation was observed (González-Moreno et al., 1997; Takenaka et al., 1997; Porchia et al., 1999). The acclimation response and the high adaptation capability of these mixotrophic microalgae are still not fully characterized.

In this study, we investigate the acclimation responses of *E. gracilis* to moderate salt stress, applying 50, 100, and 150 mM NaCl treatments to reveal how this microalga, having a common light-harvesting antenna of PSI and PSII, adapts to the stress conditions. We were interested in whether there would be a connection between the reorganization of the photosynthetic membrane and stress adaptation in *E. gracilis*. We studied the morphological and pigment composition changes, the macro-organization of photosynthetic pigment-protein complexes, and the photosynthetic energy transfer processes using various techniques, such as scanning electron microscopy (SEM) and transmission electron microscopy (TEM), small-angle neutron scattering (SANS), low-temperature fluorescence emission and circular dichroism (CD) spectroscopy, chlorophyll fluorescence induction measurements, and blue-native polyacrylamide gradient gel (BN-PAGE) analysis. In addition, we discuss the increased accumulation of paramylon.

## Materials and Methods

### Cell Culture and Growth Conditions

*Euglena gracilis* cells were grown in a medium containing 0.1 g L^−1^ from CH_3_CO_2_K, 0.1 g L^−1^ beef extract, 0.2 g L^−1^ bacto-tryptone, 0.2 g L^−1^ yeast extract, 0.02 g L^−1^ KNO_3_, 0.002 g L^−1^ (NH_4_)_2_HPO_4_, 0.001 g L^−1^ MgSO_4_ × 7H_2_O, and 0.0052 g L^−1^ CaSO_4_, supplemented with Hutner's trace elements (Hutner et al., 1966). The medium was supplemented with 50, 100, and 150 mM NaCl during salt stress treatment. The strains were grown on a rotary shaker with gentle shaking at 110 rpm at 24°C under 16 h of illumination (40 μmol photons m^−2^ s^−1^) and 8 h of darkness. The subcultures were started by setting the OD at 750 nm ~0.2. The experiments were performed for 4 days with daily sampling. The culture usually reached the stationary phase within 4–5 days.

### Chlorophyll (Chl) Determination

The pigments were extracted from the cell suspensions in 90% methanol as described previously by Devars et al. (1992) and the chlorophyll (Chl) contents were determined spectrophotometrically using the molar absorption coefficients described by Lichtenthaler and Wellburn (1983).

### Electron Microscopy

For SEM, the cells were fixed in 2.5% glutaraldehyde for 3 h in the suspension, then filtered and washed on poly-L-lysine-coated polycarbonate filters. After post-fixation in 1% OsO_4_, the samples were dehydrated in increasing ethanol concentrations, critical-point dried, covered with 15 nm gold by a Quorum Q150T ES sputter, and visualized in a JEOL JSM-7100F/LV scanning electron microscope. For TEM, cells were fixed in 2.5% (v/v) glutaraldehyde for 2 days and suspended in 2.5% tepid agar. After solidification, small cubes were cut from the samples and post-fixed in 1% (w/v) OsO_4_ for 1 h. The fixatives were buffered with 0.07 M Na_2_HPO_4_-KH_2_PO_4_ (pH 7.2). Following dehydration in aqueous solutions of increasing ethanol concentrations, the samples were embedded in Durcupan ACM resin (Fluka, Switzerland). Ultra-thin sections were cut by a Reichert Jung Ultracut M microtome (Reichert-Jung Ltd., Austria), mounted on the copper grids, then contrasted with 5% uranyl acetate and Reynold's lead citrate solution and observed by a Hitachi 7100 (Hitachi Ltd., Japan) and JEOL JEM 1011 (Jeol Ltd., Japan) TEM. To determine the repeat distance of thylakoid membranes from TEM images, we used ImageJ software. On the micrographs made with ×80 magnification, the sharp regions were selected where the thylakoid membranes appeared to be sliced perpendicularly to their membrane planes (Ünnep et al., 2014). We applied fast Fourier transformation (FFT) analysis on the selected region, which provided values on the periodicity (repeat distances—RD) of the thylakoids. This RD incorporates the widths of the two aqueous phases (the lumen and the interthylakoidal aqueous phase), and two times the width of the membrane. Each average RD was calculated from more than 160 data of stacked thylakoids, which were measured on the images of chloroplasts from 18–19 cells.

### Small-Angle Neutron Scattering (SANS)

The experiments were performed on the SANS II instrument of the Paul Scherrer Institute (PSI, Villigen, Switzerland). The applied settings for the measurements of the samples were: sample-to-detector distance (SD), 3 m; collimation, 3 m; neutron wavelength (λ), 5.52 Å. As a subtractable sample background, we measured the D_2_O-based culture medium, which was used as a suspension buffer for the algal cells during the measurements. The instrumental background was recorded with the beam blocked by a cadmium plate. All experimental data were corrected for detector efficiency obtained from the measurements performed on Cd plate, cuvette, and H_2_O in a quartz cuvette with 1 mm path length, with instrument setting of SD, 1.5 m; collimation, 2 m; and λ, 5.52 Å. For data treatment, the “Graphical Reduction and Analysis SANS Program” package (GRASP) (developed by C. Dewhurst, Institut Laue–Langevin [ILL]) was used. The observed 2D scattering patterns did not reveal significant anisotropy; therefore, further analyses were performed on 360° radially-averaged scattering curves. The scattering curves exhibited two characteristic peaks, ~0.035 and 0.06 Å^−1^. To quantify any structural changes observed with SANS, the 1D scattering curves were fitted with a sum of constant, a power function, and two Gaussian functions, similar to the method described earlier (Nagy et al., 2013). The fitting range was 0.0204–0.0856 Å^−1^. Calculated repeat distance values for the thylakoid membranes in the different samples are represented in Table 1.

**Table 1:** The calculated repeat distance (RD) values for the thylakoid membranes in the different samples.

	**Control**	**50 mM NaCl**	**100 mM NaCl**	**150 mM NaCl**
RD (Å)	188	175	181	174
	187	176	178	175

*The error calculated from the fitting of the Gaussian peaks is in most of the cases within 2 Å and in all the cases within 2.5 Å*.

### CD Measurements

Circular dichroism spectra from the control and salt-stressed cells were recorded at room temperature in the spectral range of 400–800 nm with 3 nm spectral resolution using a J815 spectropolarimeter (Jasco, Japan). The scan speed was set to 100 nm min^−1^ with an integration time of 2 s. The cells were diluted to a Chl concentration of 20 μg ml^−1^ for each sample. The measurements were carried out in standard glass cuvettes with an optical path length of 1 cm. For baseline correction, the culture media were used. The CD spectra were normalized to the Chl Qy absorption band with a reference wavelength at 750 nm. Differences of the amplitudes of (+)690 and (–)674 nm psi-type CD bands were calculated to compare the effect of salt treatment on the psi-type CD in the red spectral region.

### Measurements of Fast Chl a Fluorescence Induction Kinetics

Chl *a* fluorescence transients from the control and salt-stressed cells were measured using Aqua Pen AP-C 100 fluorometer (PSI, Czech Republic). Cultures were dark adapted for 20 min before the measurements. A cell suspension containing a total of 1 μg Chl was pipetted into a cuvette. Illumination (650 nm) was provided by an LED array which was focused on the sample to provide uniform irradiance. The fluorescence measurements were carried out with a 5 s flash of 3,000 μmol photons m^−2^ s^−1^. Fluorescence transients were recorded from the three biological repetitions.

### Determination of Electron Transport Rate and P_700_ Reduction Kinetics

The Chl *a* fluorescence and light-induced absorbance changes at 820 nm of intact cells were measured using a Dual**-**PAM**-**100 chlorophyll fluorometer. (Heinz Walz GmbH, Germany). Cell suspension equivalent to 40 μg Chl was filtered onto a Whatman glass microfibre filter (GF/C) of 25 mm diameter, which was placed in between the two microscope coverslips separated by a spacer. The basal fluorescence level (Fo) when all reaction centers open in PSII was determined first after 20 min of dark adaptation. Fm in the dark-adapted state was obtained by triggering a saturating light pulse at a light intensity of 3,000 μmol photons m^−2^ s^−1^. Actinic red light with increasing intensity was applied consecutively in the range of 10–664 μmol photons m^−2^ s^−1^ for 2 min which was enough to achieve the steady-state Fo. The Fm' level was obtained at each actinic light intensity by a saturating light pulse. Calculated parameters are represented in Table 2. ETR and redox changes of P700 were determined from the light response curves. The PSI yield (φ_I_) and PSII yield (φ_II_) were calculated according to Kramer et al. (2004) and Klughammer and Schreiber (1994), respectively.

**Table 2:** The chlorophyll (Chl) fluorescence parameters of *E. gracilis* cells grown under salt stress.

**Day**	**NaCl (mM)**	**F_**o**_**	**F_**m**_**	**F_**v**_/F_**m**_**	**NPQ**
	0	0.031 ± 0.005	0.087 ± 0.007	0.67 ± 0.03	0.43 ± 0.12
1	50	0.030 ± 0.003	0.083 ± 0.001	0.67 ± 0.03	0.41 ± 0.14
	100	0.026 ± 0.001	0.079 ± 0.003	0.67 ± 0.02	0.44 ± 0.10
	150	0.028 ± 0.004	0.087 ± 0.005	0.67 ± 0.03	0.48 ± 0.03
	0	0.027 ± 0.002	0.088 ± 0.007	0.68 ± 0.01	0.50 ± 0.04
4	50	0.027 ± 0.004	0.093 ± 0.012	0.69 ± 0.01	0.55 ± 0.10
	100	0.030 ± 0.002	0.096 ± 0.008	0.69 ± 0.01	0.54 ± 0.13
	150	0.030 ± 0.004	0.097 ± 0.011	0.69 ± 0.01	0.51 ± 0.06

*The cells were grown in the presence of the indicated NaCl concentrations. The data represent mean ± SE of three different batches of cultures*.

### Measurement of Non-photochemical Quenching (NPQ)

The protocol of Genty et al. (1989) was used to measure the non-photochemical quenching (NPQ) of Chl fluorescence. The measurements were done using a Dual-PAM-100 chlorophyll fluorometer. The dark-adapted cells were first exposed to a weak modulated beam of 2 μmol photons m^−2^ s^−1^ followed by a saturating flash of 8,000 μmol photons m^−2^ s^−1^ for a duration of 800 ms. After 20 s, the cells were exposed for 15 min to a continuous actinic light of 660 μmol photons m^−2^ s^−1^. Thereafter, the cells were exposed to a saturating pulse of 8,000 μmol photons m^−2^ s^−1^ for 800 ms. Continuous actinic light was turned off after 10 s, followed by 5 min of dark adaptation (to determine the recovery of fluorescence).

### Low-Temperature Fluorescence Emission Spectroscopy

Fluorescence emission spectra at 77 K were recorded with a Fluorolog 3 double-monochromator spectrofluorometer (Horiba Jobin-Yvon, IL, USA). The cell suspension equivalent to 5 μg Chl/ml was filtered and deposited on Whatman glass fiber (GF/B) discs. The filters were flash frozen in liquid nitrogen and transferred to a Dewar vessel filled with liquid nitrogen that was placed into the measurement chamber of the spectrofluorometer. The emission spectra from 650 to 800 nm were recorded with excitation wavelengths of 436 and 480 nm. The excitation and emission bandwidths were set to 3 and 5 nm, respectively. The measurements were performed with 1 nm resolution and 1 s integration time. Three independent repetitions were made for each treatment and averaged.

### Determination of Pigment Composition

For pigment determination, 1.5 ml of cell suspensions harvested from day 0 to day 4 were pelleted by centrifugation at 3,000 g for 3 min and snap frozen in liquid nitrogen and stored at −80°C until use. The pigments were extracted by resuspending the cells in ice-cold 100% acetone followed by 30 min dark incubation with continuous shaking at 1,000 rpm. The samples were centrifuged at 11,500 *g* for 10 min at 4°C and the supernatant was passed through a PTFE 0.2 μm pore size syringe filter.

The pigment composition of the cells was determined by high-performance liquid chromatography (HPLC), using a Shimadzu Prominence HPLC (Shimadzu Corp., Japan) system as described by Zsiros et al. (2020). The pigments were identified according to their retention times and absorption spectra and quantified by the integrated chromatographic peak area recorded at the wavelength of maximum absorbance for each kind of pigments using the corresponding molar decadic absorption coefficient (Wright et al., 2005).

### Thylakoid Isolation and Separation Using BN-PAGE

The thylakoid membranes were isolated from 4-day old cells in a medium containing 50 mM [4-(2-hydroxymethyl)-1-piperazineethanesulfonicacid] (HEPES)/KOH buffer, pH 7.5, 300 mM Sorbitol, 2 mM Na_2_-ethylenediaminetetraaceticacid (EDTA), 1 mM MgCl_2_, and 1% w/w bovine serum albumin (BSA) by breaking the cells in a Precellys Evolution (Bertin Technologies, France) homogenizer using the glass beads according to the protocol of Block and Albrieux (2018) with slight modifications. The thylakoid fractions from the disrupted cells were collected on a three-step Percoll gradient, washed with 50 mM HEPES-KOH pH 8.0 and 330 mM Sorbitol containing buffer, and solubilized with 2% (w/v) n-dodecyl-β-D-maltoside (DDM) in 25 mM bis-Tris/HCl pH 7.0, 20% glycerol (w/v) for 30 min at 4°C. The unsolubilized membranes were removed by centrifugation at 18,000 g for 20 min. The solubilized complexes were separated on a 5–13% (w/v) BN-PAGE, according to Schagger et al. (1994).

### Paramylon Extraction and Quantification

Paramylon was extracted and purified according to the protocol of Barsanti et al. (2001) with small modifications. The cells (control as well as salt-treated on day 4) were pelleted by centrifugation of 20 ml culture and frozen for a minimum of 4 h. Then, the pelleted cells were resuspended in a medium containing 1% SDS and 5% Na_2_EDTA and incubated for 30 min at 37°C. After incubation, the suspension was centrifuged for 10 min at 1,000 g to extract the paramylon granules. The treatment was repeated, and the extract was washed two times with hot distilled water (70°C). After washing, the suspension was deposited on a pre-weighted Whatman glass microfibre filter (GF/C) and dried overnight at 90°C. The dried filters were weighted to quantify the paramylon content. The amount of paramylon per milligram dry weight was calculated for all sample types. The dry weight was determined gravimetrically.

### Statistical Analyses

The Origin Pro 8 program (OriginLab Corporation, MA, USA) was used for the statistical analyses. All the measurements were carried out using at least three independent biological experiments. Data are expressed as mean ± SE. The significant levels are tested using one-way ANOVA at *p* < 0.05. The multiple comparisons of the means to determine the trends and pattern of distribution of the data were done by Tukey's *post-hoc* test.

## Results

### Effect of NaCl on the Growth Rate of *E. gracilis*

**Figure 1:**
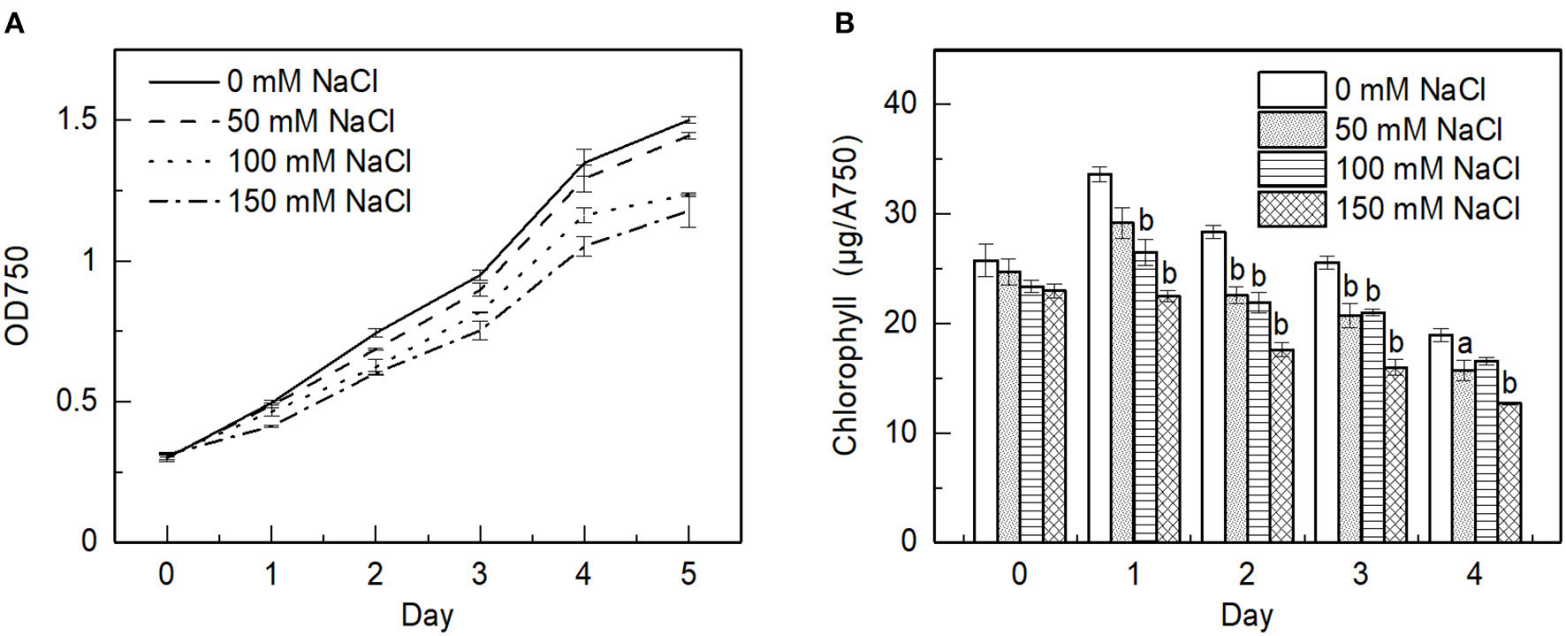
Effect of salt on the growth rate and chlorophyll (Chl) content of *E. gracilis* cells monitored over a time period of 5 days. **(A)** Representative growth curves; **(B)** Chl content normalized to the optical density. The data represent the mean ± SE of three independent experimental batches. The different letters on the bars indicate significant differences compared with the control (ANOVA with Tukey's test). a = *p* <0.05; b = *p* < 0.01.

The time course of the salt-stress driven changes of the growth rate and total Chl contents of the cells were determined spectrophotometrically. The optical density values at 750 nm for the salt treated cells compared with the control revealed a reduction in the growth rate (Figure 1A). In all cases, the cells appeared to reach the stationary phase on day 4. The total Chl content was decreased with the increasing salt concentrations (Figure 1B). These findings are in agreement with the earlier observation on salt-treated *E. gracilis* and *C. reinhardtii* cells (González-Moreno et al., 1997; Subramanyam et al., 2010; Neelam and Subramanyam, 2013).

### Structural Changes in the Macro-Organization of Pigment-Protein Complexes

**Figure 2:**
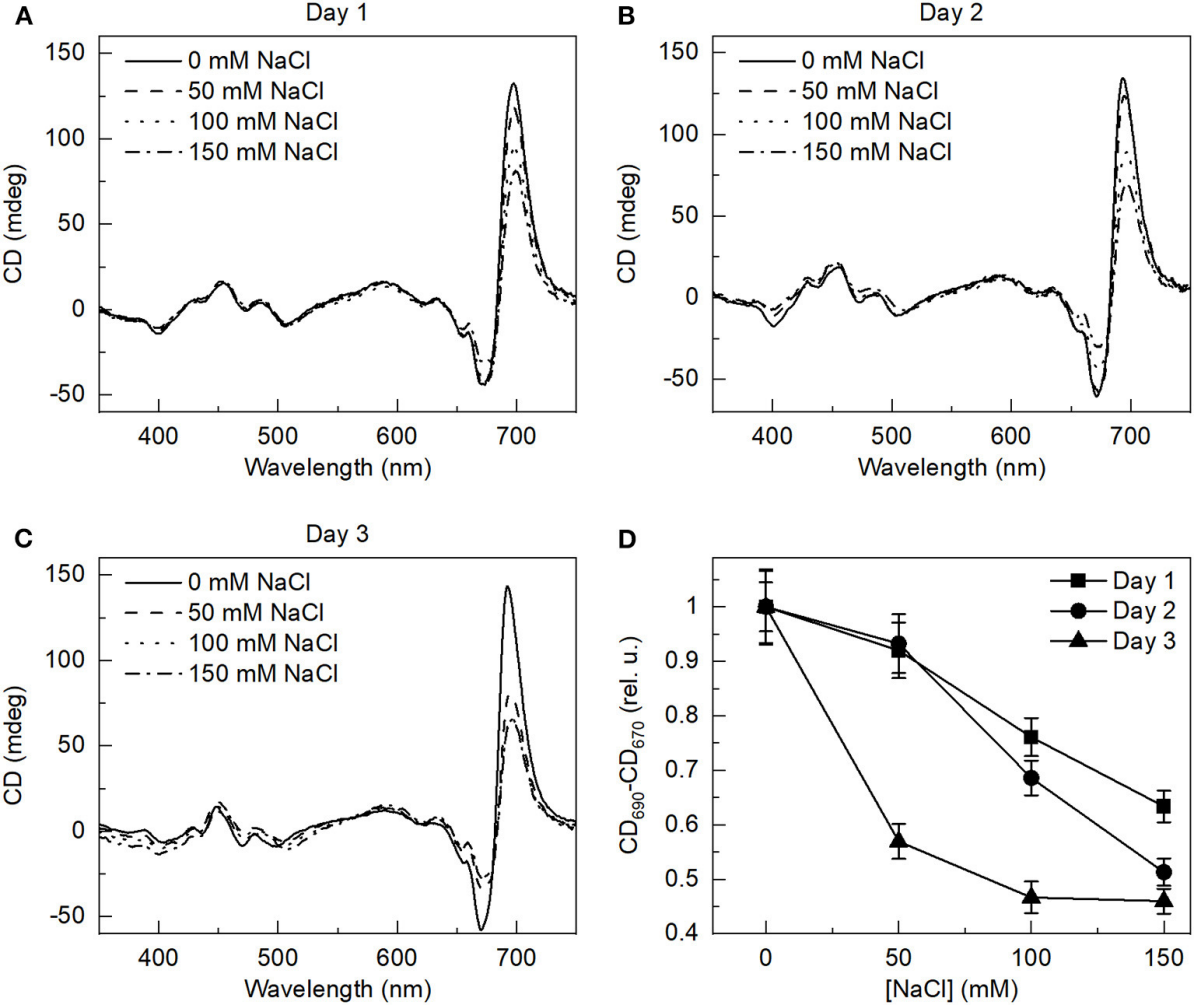
Circular dichroism spectra of the *E. gracilis* cells grown under different salinity concentrations on **(A)** day 1, **(B)** day 2, and **(C)** day 3, as indicated, and **(D)** dependence of the total amplitude of the psi-type circular dichroism (CD) bands in the red spectral region (CD690–CD670) on the salinity of the growth medium. The spectra are normalized to the red absorption maxima. The data represent mean ± SE of three independent experimental batches in panel D, the third-day salt-treated data significantly differed from the control (ANOVA with Tukey's test, *p* < 0.05).

The decreased Chl content might indicate the alterations in the photosynthetic apparatus under salt stress. Therefore, CD spectroscopy was used to monitor the changes in the macro-organization of the thylakoid membranes upon salt treatment. The CD spectra recorded from the control and salt-treated cells on different days are shown in Figure 2. In the Chl *Q*_y_ region (red maximum ~ 696 nm), the spectra showed characteristic psi-type CD signatures similar to the higher plant thylakoid membranes, originating from the long-range chiral dipole-dipole interactions of Chls of PSII and LHCII embedded in the membranes (Lambrev and Akhtar, 2019). However, unlike in higher plants, there was no psi-type CD in the blue region. The CD in the blue region was also somewhat different than in the green alga *C. reinhardtii* (Tóth et al., 2016) but similar to the diatom *Cyclotella meneghiniana* (Ghazaryan et al., 2016). The CD signal in the Soret region contains contributions from short-range, excitonic interactions between the Chls and carotenoids; therefore, the different carotenoid compositions can give different CD signals. The psi-type CD is associated with the presence of grana stacking and it disappears upon unstacking the membranes by removal of cations from the medium (Lambrev and Akhtar, 2019). However, the *E. gracilis* cells lack typical granular organization, similar to some other algae like diatoms, but they still have psi-type CD signature (Szabó et al., 2008). Upon salt treatment, the effects were only visible on the main psi-type CD band, the amplitude of the main band was reduced by the increment of salt concentration. The largest change was observed on the third day. No visible change was observed in the blue region of the spectra. The amplitude of psi-type CD band plotted as a function of salt concentration in Figure 2D, showed significant decrease on the third day of treatment.

### SANS Profiles

**Figure 3:**
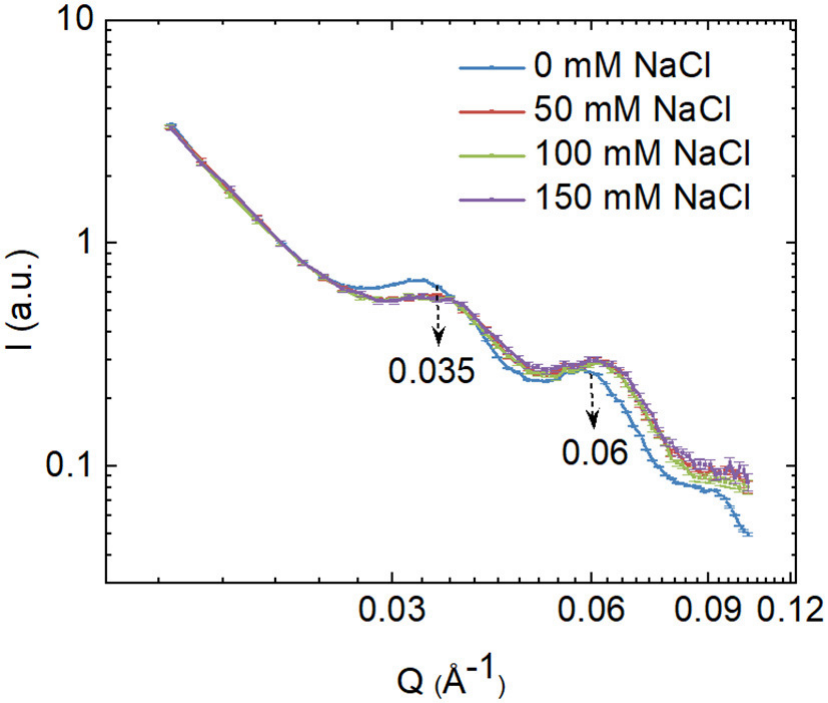
Small-angle neutron scattering (SANS) profiles of control and salt-grown *E. gracilis* cells. To facilitate the demonstration of any variation in the intensity of the peaks, the scattering curves were normalized to 1 at *Q* = 0.0204 Å^−1^, where the scattering curves followed well the power law in all the investigated samples.

We studied the ultrastructural changes of the thylakoid membranes of the *E. gracilis* cells induced by the salt treatments using SANS. The SANS profiles (Figure 3) of the cells show two well-defined peaks at ~0.035 and 0.06 Å^−1^; the 0.035 Å^−1^ peak corresponds to a repeat distance of thylakoid membranes of 179 Å; the peak at a higher q value probably originates from stacked membrane pairs (Nagy et al., 2013). Upon salt treatment, both the peaks were shifted toward higher Q-values (~ 5.5%) indicating shrinking of the periodic lamellar order. However, the shrinking effect did not show a correlation with the salt concentration, differences between the effects of different salt concentrations seem insignificant (Figure 3; Table 1).

### Morphological and Ultrastructural Changes

**Figure 4:**
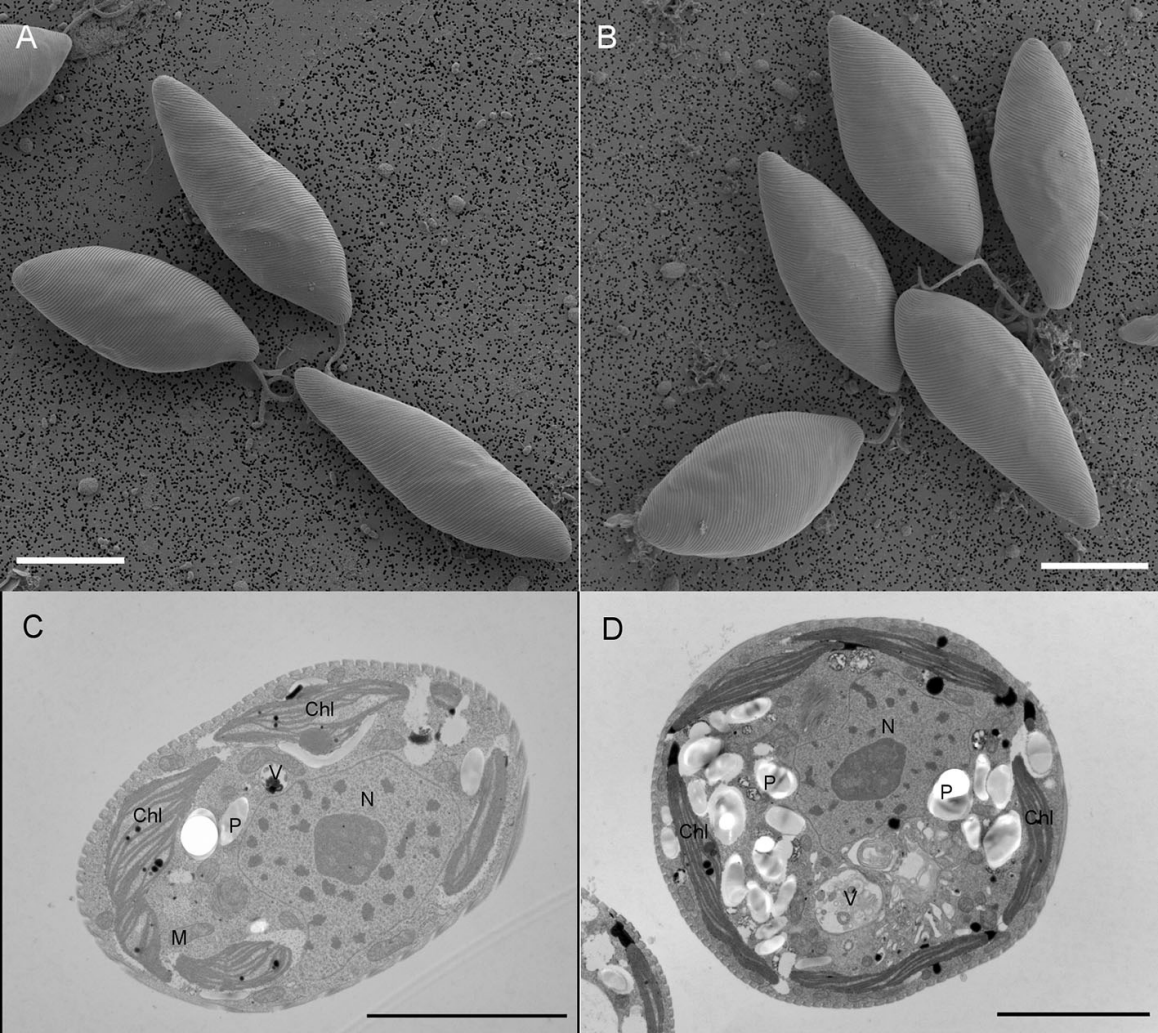
The scanning electron micrographs of **(A)** control and **(B)** 150 mM NaCl treated *E. gracilis* cells (bars: 10 μm) and the transmission electron micrographs of **(C)** control and **(D)** 150 mM NaCl treated *E. gracilis* cells (bars: 5 μm). Chl, chloroplast; P, paramylon; N, nucleus; M, mitochondrion; and V, vacuole.

We used SEM and TEM to further visualize the effects of salt-treatments on the morphology of *E. gracilis* cells and the ultrastructure of their thylakoid membranes (Figure 4). The cells did not show significant alteration in their shape and external morphology at 150 mM NaCl treatment (Figures 4A,B). The cells contained 4–5 disc-shaped chloroplasts, which were located close to the periphery of the cells, parallel to the plasma membranes (Figures 4C,D). Chloroplast comprised elongated lamellae, each formed by 2–4 (rarely 5) closely appressed thylakoids (Figures 4C,D). Upon salt-treatment, we observed shrinkage of thylakoid membranes and an increased number of paramylon grains (Figure 4D). The RD of the thylakoid membranes calculated from electron micrographs showed a reduction in the values of the salt-treated cells (18.6 ± 0.2 nm) with reference to the control (20.5 ± 0.2 nm); the shrinkage of thylakoid membranes, qualitatively, agrees well with the SANS observations.

### 77 K Fluorescence Emission Spectroscopy

**Figure 5:**
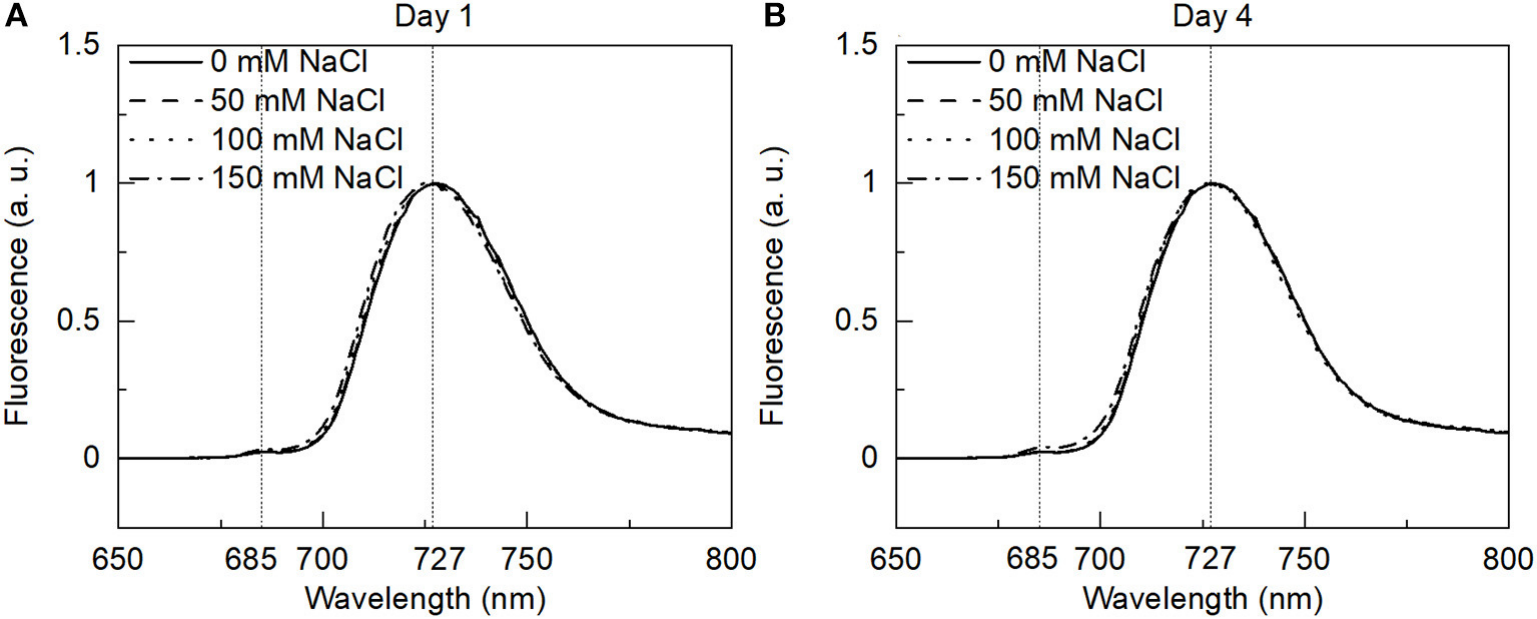
The 77 K fluorescence emission spectra of the control and salt-treated *E. gracilis* cells with 436 nm excitation were measured on **(A)** day 1 and **(B)** day 4. The spectra are normalized to their maxima.

Low-temperature fluorescence spectroscopy provides information about the distribution of excitation energy between the different pigment-protein complexes of the two photosystems. Representative fluorescence emission spectra at 77 K from the control and salt-treated cells of *E. gracilis* upon Chl *a* (436 nm) excitation are shown in Figure 5. The contributions of the two photosystems to the fluorescence can be estimated based on the emission spectra. The emission spectra are very similar to the previously published data (Doege et al., 2000) on the same species dominated by the emission from PSI only, with the peak at 727 nm. The small shoulder could also be seen at 685 nm indicating negligible fluorescence from PSII. The emission spectra are very different from the higher plant thylakoid membranes (Walters and Horton, 1995) and other green algae (Kramer et al., 1985), where both the photosystems display distinct peaks. Similar results were obtained upon 480 nm excitation (preferentially exciting Chl *b*) (data not shown). While comparing different salt treatments, no significant changes were observed, indicating no or negligible effect of salt on the photosynthetic energy transfer.

### Chl *a* Fluorescence (OJIP Transient) Analysis

**Figure 6:**
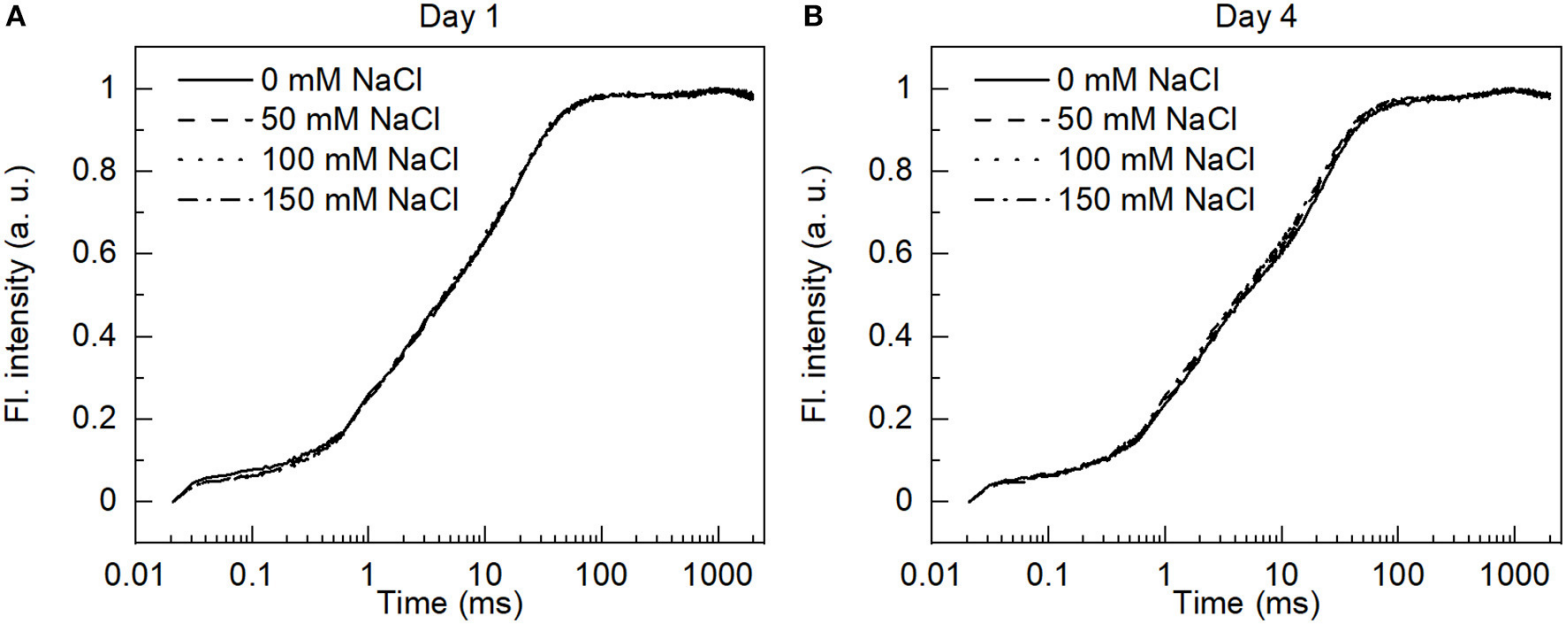
Variable Chl *a* fluorescence induction curves of control and salt-treated dark-adapted cells (20 min) recorded on **(A)** day 1 and **(B)** day 4 using 650 nm saturating light.

The fast Chl *a* fluorescence induction (OJIP) transients, measured by exposing the dark-adapted samples to high light, show a complex multistep rise curve which represents a fingerprint of the species and the physiological status of the cells (Srivastava and Strasser, 1995). The shape of transient is sensitive to many photosynthetic processes, excitation energy flow from the antenna to the reaction centers, the structural plasticity of the reaction center complexes, and the electron transfer on the donor and the acceptor side of PSII, and the availability and redox state of intersystem electron carriers and downstream electron transfer to PSI. According to the theory of energy flux, any change in any of these processes will induce a change in the shape of the induction curve. The fluorescence induction curves recorded from *E. gracilis* cells have an unusual shape, with no well-visible J and I steps (Figure 6). In the higher plants (Stirbet et al., 2018) and green alga *C. reinhardtii* (Kodru et al., 2015), after 100 ms, there is a fast drop in the transient fluorescence which is usually assigned to the state transitions, NPQ or redox state of P700 (Strasser et al., 2004). In the case of *E*. *gracilis*, the induction curve stays flat up to 1000 ms. This could be very well explained by the common antenna system of PSI and PSII or the absence of the classical violaxanthin cycle. When comparing the transients for different salt-treatments, no remarkable difference could be observed (Figure 6). Very similar F_v_/F_m_ values, 0.6 were recorded for both the salt-treated and control cells, thus the functional state of PSII is not affected by the salt treatment.

### PSII Photochemical Efficiency and Quenching Analysis

**Figure 7:**
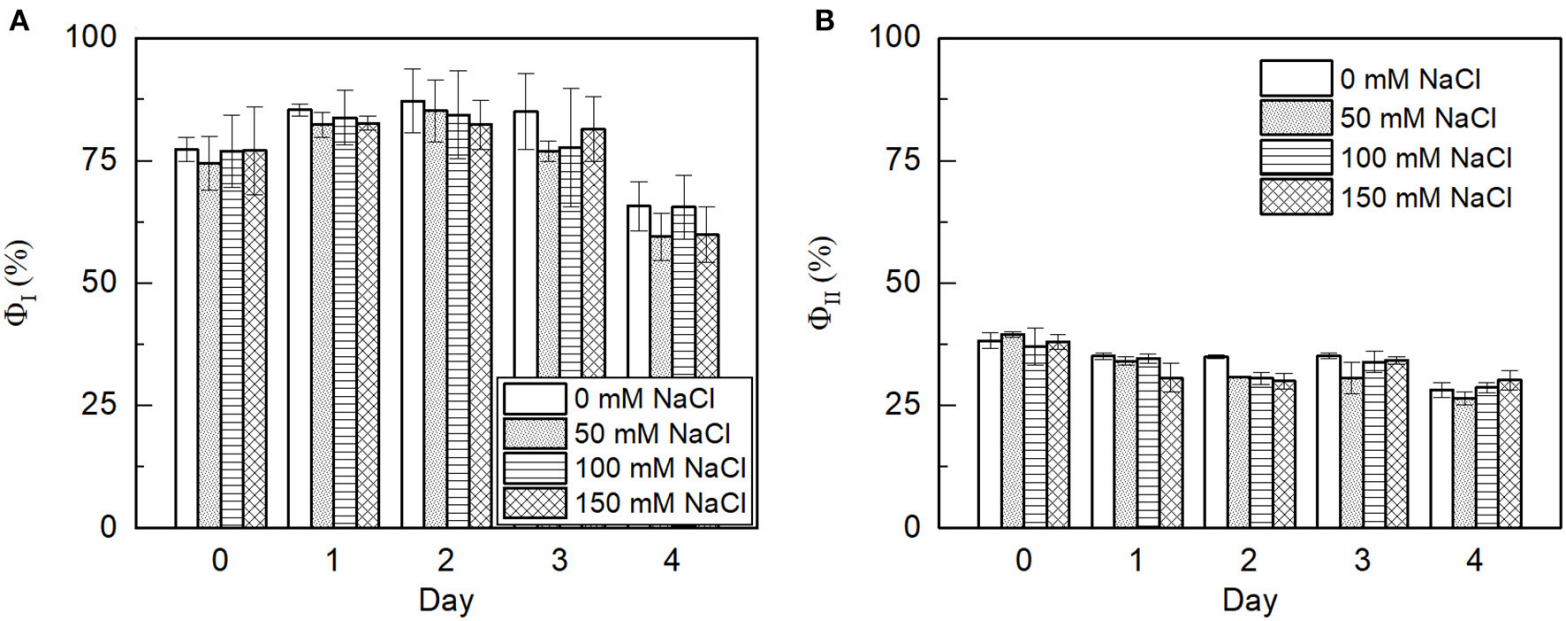
The photosynthetic fluorescence parameters of *E. gracilis* cells grown under saline stress for 4 days. **(A)** Photosystem I (PSI) yield and **(B)** photosystem II (PSII) yield of the cells subjected to 0 (control), 50, 100, and 150 mM NaCl salt treatments at 220 μmol photon m^−2^ s^−1^ of the light response curve. Data represent mean ± SE of three independent cultures and shows no significant differences in the means.

Furthermore, to understand how salt stress affects photosynthesis in *E. gracilis* cells, the light curves were recorded. ETR and PSI activity in both the control and salt-treated cells were measured on different days (Figure 7). In good agreement with the previous observations, that upon NaCl treatment there is no significant effect on the functional state of PSII (F_v_/F_m_) of the cells, we also found that the linear ETR and the activity of PSI were not affected. Upon irradiation with high-light intensity, both the control and salt-treated cells displayed considerable induction of non-photochemical quenching. The NPQ of 0.4 was observed which is similar to the findings of González-Moreno et al. (1997). However, the NPQ of the salt-treated cells was not significantly different from the control cells as represented in Table 2.

### P700 Reduction Kinetics

**Figure 8:**
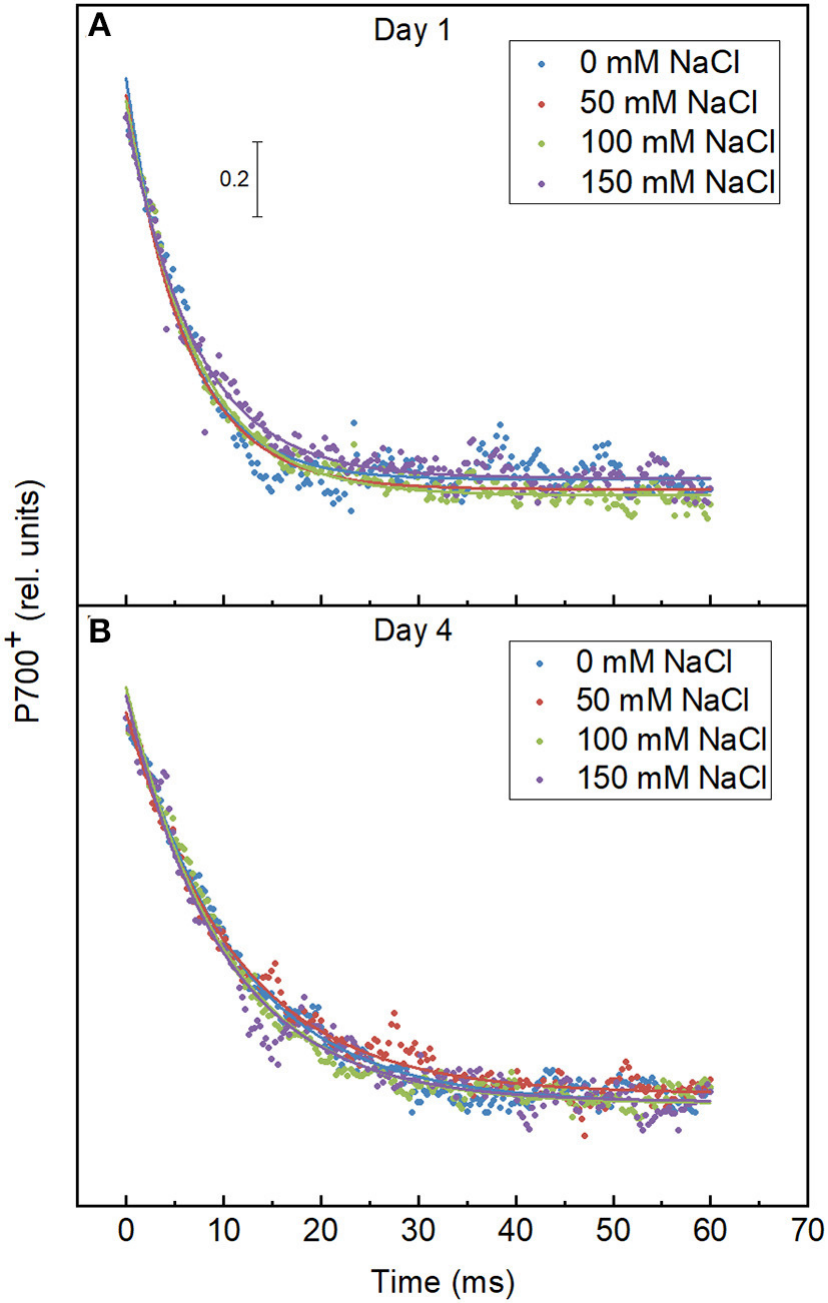
Effect of the salt treatment on the re-reduction kinetics of P700. The samples were pre-illuminated for 20 min with 530 μmol photon m^−2^ s^−1^ and re-reduction kinetics of P700 were recorded after cessation of light. The measurements were done with the *E*. *gracilis* cells grown in the indicated salt concentrations on **(A)** day 1 and **(B)** day 4.

The reduction kinetics of P700 in both the control and salt-treated cells are shown in Figure 8. The activity of PSI was monitored by the absorbance change at 830 nm due to the oxidation of P700. The fluorescence decay lifetimes of the 50, 100, and 150 mM NaCl treated cells are 11.05 ± 0.238, 10.12 ± 0.177, and 9.997 ± 0.276, which is similar to the lifetime of control cells, 10.98 ± 0.227 on day 4 of the treatment as shown in Table 3. The salt treatment induced no significant changes in the PSI activity.

**Table 3:** Lifetimes were obtained by fitting the fluorescence decay curves of P700 reduction kinetics calculated by one exponential function from the control and salt-treated cells.

**T**	**0 mM NaCl**	**50 mM NaCl**	**100 mM NaCl**	**150 mM NaCl**
Day 1	5.806 ± 0.196	6.247 ± 0.117	6.957 ± 0.116	7.092 ± 0.178
Day 4	10.98 ± 0.227	11.05 ± 0.238	10.12 ± 0.177	9.997 ± 0.276

### Assessment of the Carotenoid Content

**Figure 9:**
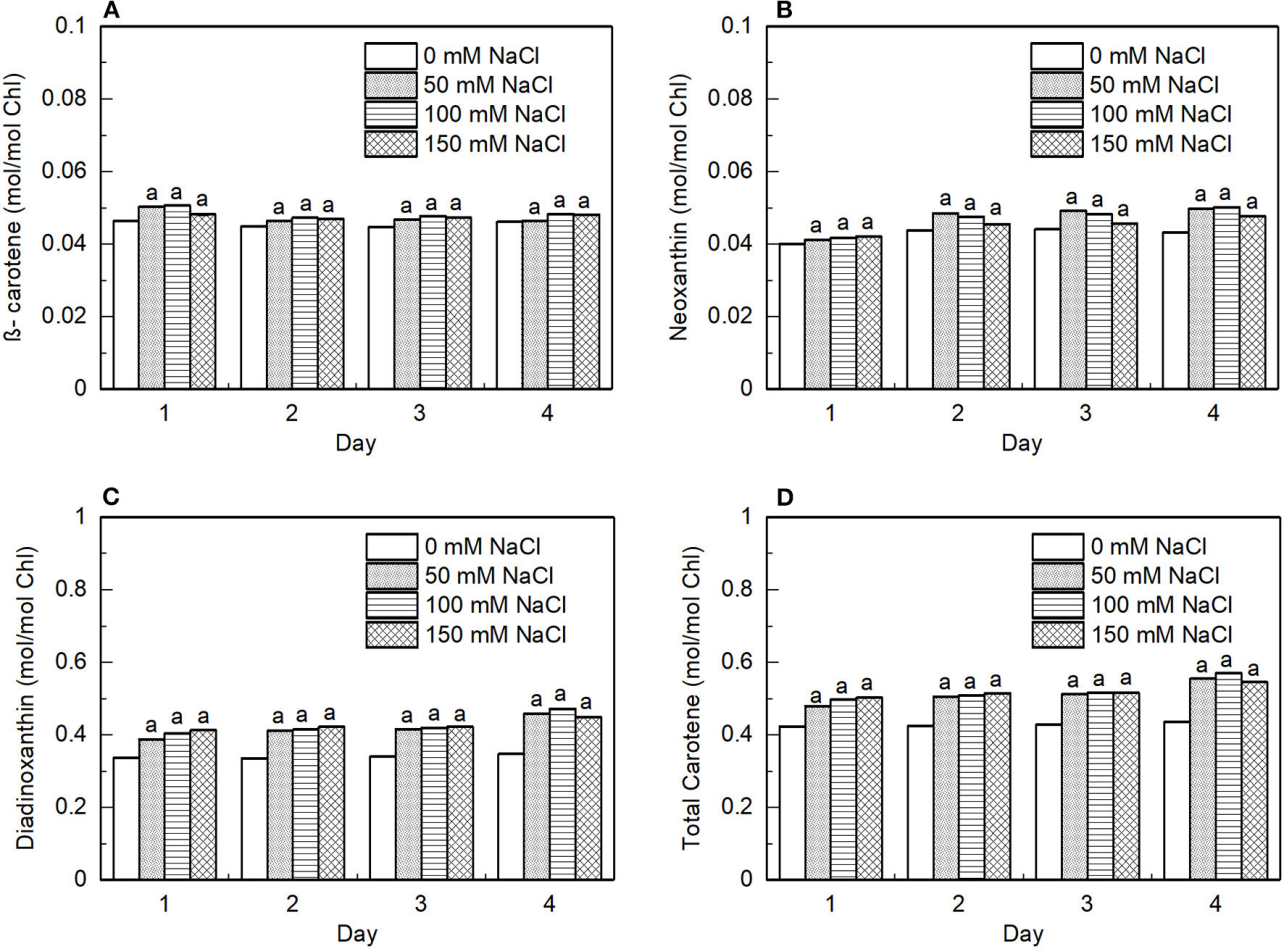
Effect of the salt-treatment on the content of photosynthetic pigments in the *E. gracilis* cells. The cellular content of **(A)** β-carotene, **(B)** neoxanthin (Nx), **(C)** diadinoxanthin (Ddx), and **(D)** total carotenoid relative to the total content of Chl in the algal cells subjected to 50, 100, and 150 mM NaCl salt treatments for 4 days. The data represent mean ± SE of three different batches. The letters indicate significant differences between the means related to the control (ANOVA with Tukey's test; *p* < 0.05).

The pigment extracts of the control and salt-treated cells were analyzed by HPLC to determine the effects of salt on the pigment composition. Typical HPLC profiles exhibit peaks for Chl *a*, Chl *b*, β-carotene, Nx, and Ddx. Ddx is found in the antenna of many diatom species (Lavaud et al., 2003), while the antenna of green algae (Goss and Jakob, 2010) and higher plants are known to contain lutein instead (Liu et al., 2004). However, diatoxanthin which is a constituent of classical Ddx-cycle in the diatoms was not detected. During salt stress, the relative composition of carotenoids did not change, however, every carotenoid alone and so the total carotenoid content increased significantly compared with Chl (Figure 9).

### Analyses of Photosynthetic Pigment-Protein Complexes by BN-PAGE

Useful information about the composition and interactions of the protein complexes in the thylakoid membrane can be obtained by solubilizing the membrane by the mild detergent treatment followed by their separation on a polyacrylamide gel. BN-PAGE is one of the methods used for the separation of protein complexes in their native and functional form. In this study, BN-PAGE was used to determine whether the salt treatment induces changes in the interactions between the protein complexes or leads to the formation of larger supercomplexes. The patterns of the thylakoid membrane protein complexes, after solubilization with DDM and separation by BN-PAGE (Supplementary Figure 1), showed similarity to that of the higher plants (Järvi et al., 2011). No visible changes were observed in the pattern of BN-PAGE when comparing different salt treatments.

### Accumulation of Paramylon Under Salt Stress

Our ultrastructural analyses indicated the accumulation of paramylon grains in the cytoplasm of stressed cells (Figure 4D). A protocol has been used to isolate paramylon (Supplementary Figure 2) to quantify its content in the different samples. Table 4 shows the changes in paramylon content at a cellular level in the *E. gracilis* control and salt-treated cells after 4 days of treatment. The accumulation of paramylon increased with the increasing salt treatment with the highest levels of paramylon observed in the cells grown in the presence of 150 mM NaCl. The increase was 2.5-, 3.5-, and 5-fold in the 50, 100, and 150 mM NaCl treated cells, respectively.

**Table 4:** Effect of salt stress on paramylon accumulation in *E. gracilis*.

**NaCl (mM)**	**PDW/CDW (μg/mg)**	**Paramylon (%)**
0	51.23 ± 6.29	100
50	126.18 ± 20.86	245.46 ± 13.36
100	181.64 ± 20.06	355.02 ± 10.6
150	261.04 ± 20.97	515.04 ± 76.67

*The cells were cultured in a medium containing different salt concentrations. After 4 days of treatment, the cells were harvested and paramylon content was determined. The experiments were done three times. CDW, cell dry weight; PDW, paramylon dry weight*.

## Discussion

The production of bioactive compounds by a microalgal cell is of high interest for biotechnological applications. For example, salt stress can induce the production of bioactive molecules; notwithstanding, salt stress can severely affect the growth and cellular metabolism of microalgae. Salt stress is also one of the stress factors that photosynthetic organisms, especially but not only in the aquatic environment, have to face. To assess the impact of salinity stress on the growth of *E. gracilis*, the optical density and the Chl content of *E. gracilis* batch culture were followed (Figure 1). We found that the growth was hindered and, in the meantime, the Chl content slightly decreased due to the stress. This is in good agreement with the observations obtained from the plant and other algal cultures stressed by salt (Zakery-Asl et al., 2014; Forieri et al., 2016; Ji et al., 2018). It was shown that salt stress could delay the transitions between the different growth phases of *Scenedesmus obliquus* (El-Katony and El-Adl, 2020). Although, we observed a slower growth rate in the salt-treated cultures, all the cultures reached the stationary phase on the fifth day. In general, salt stress could increase oxidative stress as a consequence of the degradation of Chls (El-Katony and El-Adl, 2020; Elloumi et al., 2020). It was also suggested that the Chl degradation was due to some enzymatic activity (Ji et al., 2018).

*E. gracilis* is able to tolerate relatively high salt concentration for a couple of days, however over 10 g L^−1^ concentration of NaCl caused a pronounced loss in motility and velocity (Richter et al., 2003). Investigation of the salt stress on the detergent pre-treated *E. gracilis* and isolated pellicular structures suggested that the microtubules of the pellicular strips were dissolved above 150 mM NaCl concentration, which influenced the cell movement (Murata and Suzaki, 1998). Upon stress conditions, the *E. gracilis* cells underwent morphological changes and often rounded cells were observed (Azizullah et al., 2012; Peng et al., 2015). However, we could not detect the round cells (Figure 4) even at the highest salt concentration (150 mM), although, we observed the morphological changes in the ultrastructure of the stressed cells compared with the control.

One of these changes was in the ultrastructural organization of the thylakoid membrane system (Figures 4C,D). The calculated RD of the thylakoid membranes showed reduction due to salt treatment, which was in correlation with the *in vivo* observation by SANS (Figure 3; Table 1). Gonzalez-Moreno and co-workers just assumed an increase in the stacking of *E. gracilis* chloroplast because of the change in the Chl *a* and *b* ratio (González-Moreno et al., 1997); now we have evidence for the more tightly organized thylakoid system upon the salt-treatment of *E. gracilis* cells. The salt stress induced a reduction in the RD values of the thylakoids (Figure 4D). There was a difference between the RD values of the SANS measurements and that of the TEM images. A similar difference was also observed in the Cr-treated *Chlorella variabilis* cells (Zsiros et al., 2020). The difference can be explained by the different sample preparation processes; SANS provided information about the periodic organization of the thylakoid membranes gained *in vivo* without any fixation or staining but in D_2_O instead of H_2_O (Nagy et al., 2011).

The thylakoid membrane systems of the photosynthetic organisms are capable of responding to the rapidly changing environmental conditions (Ünnep et al., 2014), however, the molecular background of this phenomenon is less known in stress acclimation processes. The light induced reversible RD changes were observed by investigating various photosynthetic organisms (Nagy et al., 2011). It was suggested that the membrane reorganization changes observed by SANS were associated with efficient NPQ (Nagy et al., 2012; Ünnep et al., 2020).

The CD measurements also indicated reorganizations of the thylakoid membrane. The CD spectra revealed discernible changes in the macro-organization of the protein complexes in the thylakoid membranes (Figure 2). The CD spectrum of *E. gracilis* cells differed from that of plant or *C. reinhardtii* thylakoids (Tóth et al., 2016; Lambrev and Akhtar, 2019), this might be related to differences in the light-harvesting antenna and pigment compositions. Interestingly, the amplitude of the psi-type CD band, which is generally related to the grana stacking and its intensity depends on the extent of long-range chiral order, the domain size, and the direction of the chiral order (Keller and Bustamante, 1986; Kim et al., 1986), decreased upon the elevated salt concentration. The other part of the spectrum showed only minor changes. It is known that the chirally organized macrodomains show structural flexibility in the photosynthetic membranes, apparently providing photoprotective capability at the supramolecular level (Garab et al., 1988; Barzda et al., 1996; Ünnep et al., 2020). The thylakoids of diatom *Phaeodactylum tricornutum* have been shown to be arranged into loosely stacked, multilamellar membrane system, without having strictly distinguishable granal and stromal regions, but still exhibiting the intense psi-type CD signals (Szabó et al., 2008) that showed sensitivity to the light, temperature, and osmolarity. We observed a similar structure in the *E. gracilis* cells. Whereas the CD spectra indicated salt-stress induced alterations in the macro-organization of the photosynthetic complexes, the BN-PAGE did not reveal alterations in the pattern of these pigment-protein complexes (Supplementary Figure 1). The changes in the RD values and in the psi-type CD signals suggest an alteration in the supramolecular array of the complexes in association with the changes in the membrane ultrastructure. The composition of the photosynthetic complexes and the organization of the thylakoid membrane of the *E. gracilis* cells differ from higher plants and other well-studied microalgae, therefore more detailed investigation of the molecular mechanism behind the observed changes are needed, but these are out of the scope of this study.

One of the main differences of *E. gracilis* from higher plants is that both the photosystems use a common antenna system consisting of LHCI and LHCII proteins; and, in contrast with the higher plants, the energy-dependent quenching (qE) in *E. gracilis* is independent of the xanthophyll cycle and the aggregation of LHCII (Doege et al., 2000). The 77 K fluorescence spectra of *E gracilis* cells also reflect the specific antenna composition usually showing one maximum at ~722 nm (Tschiersch et al., 2002). The characteristic fluorescence band of PSII at ~683 nm was shown to become visible only when the cells were treated with norflurazon that caused a reduction in the carotenoid content and drastically diminished the amount of the common antenna and the originally little amount of LHCII trimer (Tschiersch et al., 2002). Based on the 77 K fluorescence spectra, the salt treatment did not induce significant changes in the photosynthetic energy transfer (Figure 5) which might reflect that there were no significant changes in the amount of the photosynthetic complexes either, proved with the BN-PAGE analyses (Supplementary Figure 1).

At higher salt concentrations, the osmotic stress and the toxic ionic stress of salt can reduce the photosynthetic efficiency. The effect on the photosynthetic efficiency is often related to the damage of PSII due to the production of ROS. It was shown that NaCl stress can cause the damage of the oxygen evolving complex and the PSII reaction center leading to the suppression of the electron transport at the donor and acceptor sides influencing the light energy application (Ji et al., 2018). The OJIP curves differed in the cases of *E. gracilis* and in higher plants, which may be due to the differences in their antenna systems. The OJIP curves did not change significantly by the salt treatments (Figure 6), indicating that the treatments did not modify the light-dependent photosynthetic processes. This was corroborated by our observation regarding the functional state of PSII (F_v_/F_m_), the ETR, and the activity of PSI all that were not affected by the NaCl treatments (Figures 7, 8; Table 2). Prior to the measurements, the samples were set to the same Chl concentration, however, the Chl content was slightly decreased to the cell mass in the salt treated cultures, therefore the results reflected the functionality of the photosynthetic pigment protein complexes, which were not affected by the salt treatment. Interestingly, it was earlier reported that the reactivated *E. gracilis* cells could show enhanced Chl accumulation and decreased photosynthetic efficiency when they were grown in an acidic organotrophic medium with glutamate and malate as carbon source supplemented with 200 mM NaCl (González-Moreno et al., 1997). It must be noted that when 100 mM NaCl was applied alone as a stress factor, the Chl content was decreased and the changes of the photosynthetic parameters were not as pronounced. This might suggest that the adaptation of the mixotrophic *E. gracilis* cells to moderate salt stress depends on the pretreatment of the cells and on the composition of the culture media. Furthermore, the salt stress besides the photosynthesis could affect aerobic respiration, too, and so could the cell growth; since biomass accumulation depends on the balance of photosynthesis and respiration (Jacoby et al., 2011). In addition, photosynthetic output depends on the function of ribulose-1, 5-bisphosphate carboxylase/oxygenase (Rubisco) that can switch between the release or incorporation of CO_2_ (Wingler et al., 2000). Salt stress can also affect the activity of Rubisco this way influencing biomass production (Wingler et al., 2000; He et al., 2014). Further investigations are needed to reveal how exactly the salt stress affected the respiration or Rubisco activity in *E. gracilis*.

We observed significant changes in the pigment composition of the cells, upon salt treatment, the amount of carotenoids normalized to Chl increased significantly (Figure 9D). The carotenoids in photosynthetic organisms can participate in light-harvesting and at the same time, they have an important role in the photoprotective mechanisms and could serve as antioxidants, can modulate the membrane microviscosity, and participate in the maintenance of proper cellular architecture (Domonkos et al., 2013). The increased carotenoid content might protect the lipids from oxidation; thus, protecting the function of the photosynthetic complexes.

Importantly, the paramylon content of the cells was increased drastically (Table 3). The accumulation of the paramylon granules (Supplementary Figure 2) was clearly visible on the TEM pictures (Figure 4D). In the *E. gracilis* cells, paramylon serves as a storage form of sugar. Paramylon is a β-1,3-glucan, that has high biotechnological potential and was considered as a functional food (Nakashima et al., 2018; Barsanti and Gualtieri, 2019). Several potential biomedical applications of paramylon, such as immunostimulant and an anti-inflammatory agent are under thorough investigations (Nakashima et al., 2017; Okouchi et al., 2019), and it can serve as a raw material for biofuels, as well (Inui et al., 1982; Zimorski et al., 2017; Khatiwada et al., 2020). The paramylon content decreased upon 200 mM NaCl treatment because it was degraded and converted to soluble sugars, causing trehalose accumulation in the cells (Takenaka et al., 1997). Trehalose accumulation was also increased by KCl, NaCl, sugars, and sugar alcohols indicating that the osmolarity of the medium induced the degradation of paramylon (Takenaka et al., 1997). Porchia et al. (1999) found trehalose accumulation upon the salt stress administered in the late exponential phase. On contrary, we observed an accumulation of paramylon during moderate salt stress. However, we examined and treated cultures in the exponential growth phase. We can assume that this storage sugar can play an important role in the adaptation mechanism and can enable a rapid response of the cells when facing osmotic stress. It seems that the *E. gracilis* cells can compensate for the moderate salt stress, by reorganization of the thylakoid membrane and adjustment of its metabolism, without having a severe effect on other cell functions.

From the results, we can conclude that *E. gracilis* cells can maintain the photosynthetic activity with modification of the pigment composition and the reorganization of the thylakoid membranes; further, the increment of the concentration of paramylon prepares the exponentially growing cells for a higher osmotic shock.

## Data Availability Statement

The raw data supporting the conclusions of this article will be made available by the authors, without undue reservation.

## Author Contributions

BU and SK conceived the study. SK performed the 77 K fluorescence measurements. SK and LK investigated the photosynthetic activity and determined the pigment compositions. TK and ÁD participated in cell culturing and treatments. ID and KS did the SEM and TEM analyses, respectively. The quantitative analyses of the TEM images were performed by ID and SK. The paramylon content determination and the BN-PAGE were performed by ID joined by SK. GN, RÜ, KS, OZ, and GG participated in the SANS measurements. LS, KB, and SN participated in the data analyses. The paper was written by BU, LS, SK, ID, KS, and GG with all authors contributing to the writing and finalization of the manuscript. All authors contributed to the article and approved the submitted version.

## Funding

This work was supported by the grants of the Hungarian Ministry for National Economy and the National Research Development and Innovation Office of Hungary (GINOP-2.3.2-15-2016-00058 to BU, OTKA K 128679 to GG, and OTKA FK124748 to KS) and of the Czech Science Foundation (GA CR 19-13637S to GG). KS is also grateful for the support of the János Bolyai Research Scholarship of the Hungarian Academy of Sciences. A portion of this research used resources at the Spallation Neutron Source, a DOE Office of Science User Facility operated by the Oak Ridge National Laboratory.

## Conflict of Interest

LS was employed by the company Szilak Laboratories Ltd. TK and ÁD were employed by the company Division for Biotechnology, Bay Zoltán Nonprofit Ltd. for Applied Research. The remaining authors declare that the research was conducted in the absence of any commercial or financial relationships that could be construed as a potential conflict of interest.

## Publisher's Note

All claims expressed in this article are solely those of the authors and do not necessarily represent those of their affiliated organizations, or those of the publisher, the editors and the reviewers. Any product that may be evaluated in this article, or claim that may be made by its manufacturer, is not guaranteed or endorsed by the publisher.

## Supplementary Material

Supplementary Figure 1 The thylakoid membrane protein complexes in *E. gracilis* separated on a 5–13% blue-native polyacrylamide gradient gel (BN-PAGE). **(A)** BN pattern of pigment-protein complexes in the control and salt-treated cells. **(B)** The BN gel after Coomassie brilliant blue (G-250) staining. The samples loaded onto each gel lane contained 8 μg Chl.

Supplementary Figure 2 The scanning electron micrograph of paramylon granules isolated from 150 mM NaCl treated *E*. *gracilis* cells. Bar 1 μm.

## Abbreviations

Chl: chlorophyll
CD: circular dichroism
Ddx: diadinoxanthin
EDTA: ethylenediaminetetraaceticacid
ETR: electron transport rate
HPLC: high-performance liquid chromatography
LHCI: light harvesting complex-I
LHCII: light-harvesting complex II
NPQ: non-photochemical quenching
PAGE: polyacrylamide gel electrophoresis
PSI: photosystem I
PSII: photosystem II
RD: repeat distance
ROS: reactive oxygen species
Rubisco, ribulose-1: 5-bisphosphate carboxylase/oxygenase
SANS: small-angle neutron scattering
SEM: scanning electron microscopy
TEM: transmission electron microscopy
DDM: n-dodecyl-β-maltoside
FFT: fast-Fourier transformation
HEPES: [4-(2-hydroxymethyl)-1-piperazineethanesulfonicacid].
