# Supplementary figures and images for: Salt Stress Induces Paramylon Accumulation and Fine-Tuning of the Macro-Organization of Thylakoid Membranes in Euglena gracilis Cells
Source: Front Plant Sci. 2021 Nov 16;12:725699. doi: 10.3389/fpls.2021.725699 (PMC8636990; doi:10.3389/fpls.2021.725699)

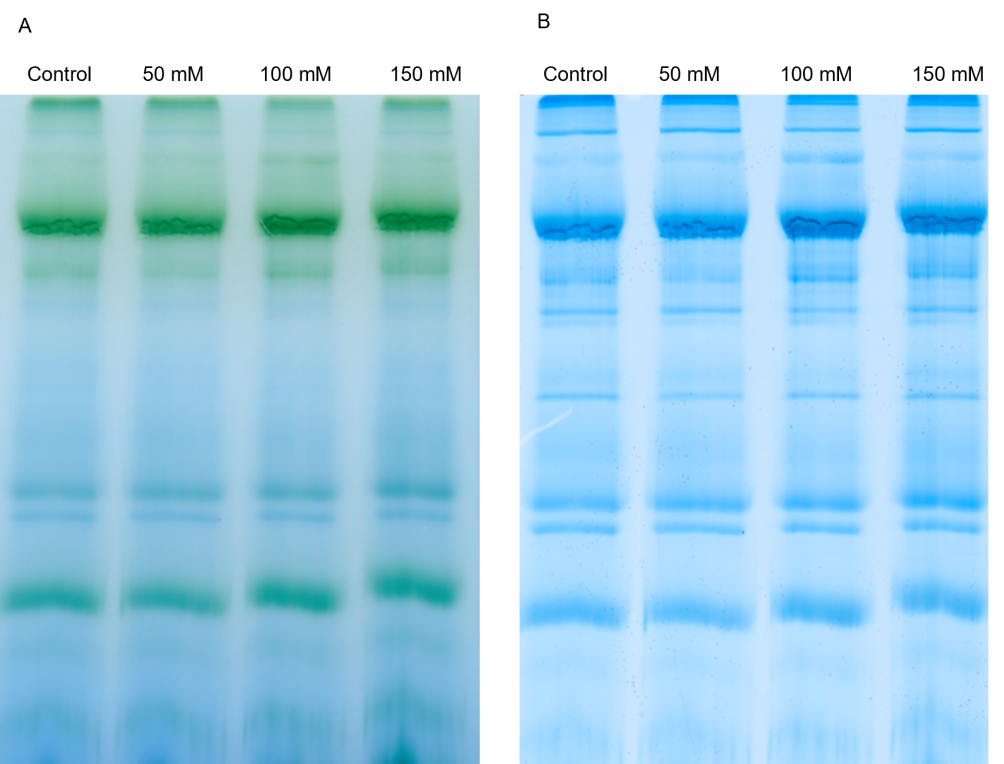

Supplement: Supplementary Figure 1 — The thylakoid membrane protein complexes in E. gracilis separated on a 5–13% blue-native polyacrylamide gradient gel (BN-PAGE). (A) BN pattern of pigment-protein complexes in the control and salt-treated cells. (B) The BN gel after Coomassie brilliant blue (G-250) staining. The samples loaded onto each gel lane contained 8 μg Chl. [file Image_1.TIF]

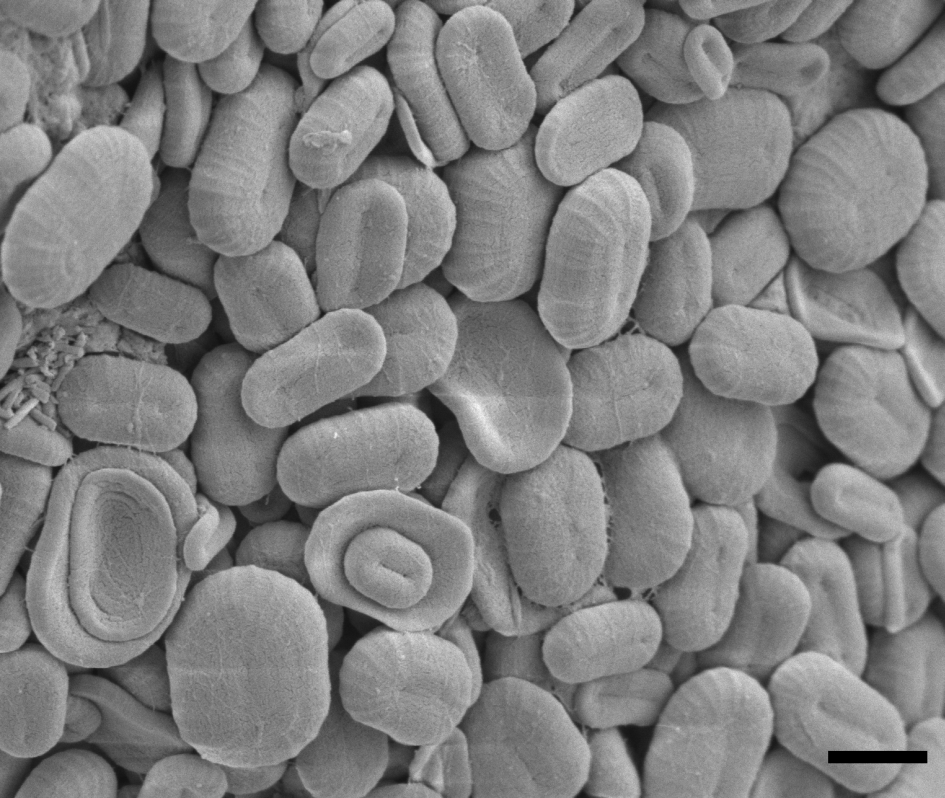

Supplement: Supplementary Figure 2 — The scanning electron micrograph of paramylon granules isolated from 150 mM NaCl treated E. gracilis cells. Bar 1 μm. [file Image_2.TIF]
